# The effect of optimised patient information materials on recruitment in a lung cancer screening trial: an embedded randomised recruitment trial

**DOI:** 10.1186/s13063-018-2896-9

**Published:** 2018-09-18

**Authors:** Adwoa Parker, Peter Knapp, Shaun Treweek, Vichithranie Madhurasinghe, Roberta Littleford, Stephanie Gallant, Frank Sullivan, Stuart Schembri, Jo Rick, Jonathan Graffy, David J. Collier, Sandra Eldridge, Anne Kennedy, Peter Bower

**Affiliations:** 1 York Trials Unit, Department of Health Sciences, University of York, York, YO10 5DD UK; 2 Department of Health Sciences, Seebohm Rowntree Building, University of York, York, YO10 5DD UK; 3 Health Services Research Unit, University of Aberdeen, 3rd Floor, Health Sciences Building, Foresterhill, Aberdeen, AB25 2ZD UK; 4 Blizard Institute, Barts and The London School of Medicine and Dentistry, 4 Newark Street, London, E1 2AT UK; 5 Tayside Clinical Trials Unit, University of Dundee, Dundee, UK; 6 Centre for Public Health Nutrition Research, University of Dundee, Dundee, UK; 7 School of Medicine, Medical & Biological Sciences, North Haugh, St Andrews, UK; 8 School of Medicine, University of Dundee, Ninewells Hospital & Medical School, Dundee, DD1 9SY Scotland, UK; 9 MRC North West Hub for Trials Methodology Research, Manchester Academic Health Science Centre, University of Manchester, Williamson Building, Manchester, M13 9PT UK; 10 Department of Public Health and Primary Care, The Primary Care Unit, University of Cambridge, Cambridge, CB2 OSR UK; 11 William Harvey Research Institute, Barts and the London Queen Mary University of London, Charterhouse Square, London, EC1M 6BQ UK; 12 NIHR Collaboration for Leadership in Applied Health Research and Care (CLAHRC) Wessex, Health Sciences, University of Southampton, Highfield, Southampton, SO17 1BJ UK; 13 Centre for Academic Primary Care, School of Social and Community Medicine, University of Bristol, 39 Whitely Road, Bristol, BS8 2PS UK

**Keywords:** Recruitment, Patient information, Research methodology, Randomised controlled trial, Study within a trial (SWAT)

## Abstract

**Background:**

Written participant information materials are important for ensuring that potential trial participants receive necessary information so that they can provide informed consent. However, such materials are frequently long and complex, which may negatively impact patient understanding and willingness to participate. Improving readability, ease of comprehension and presentation may assist with improved participant recruitment. The Systematic Techniques for Assisting Recruitment to Trials (MRC START) study aimed to develop and evaluate interventions to improve trial recruitment. This study aimed to assess the effectiveness of an optimised participant information brochure and cover letter developed by MRC START regarding response and participant recruitment rates.

**Methods:**

We conducted a study within a trial (SWAT) embedded in the EarlyCDT Lung Cancer Scotland (ECLS) trial that aimed to assess the effectiveness of a new test in reducing the incidence of patients with late-stage lung cancer at diagnosis compared with standard care. Potential participants approached for ECLS were randomised to receive the original participant information brochure and accompanying letter (control group) or optimised versions of these materials which had undergone user testing and a process of re-writing, re-organisation and professional graphic design (intervention group). The primary outcome was the number of patients recruited to ECLS. The secondary outcome was the proportion of patients expressing an interest in participating in ECLS.

**Results:**

In total, 2262 patients were randomised, 1136 of whom were sent the intervention materials and 1126 of whom were sent the control materials. The proportion of patients enrolled and randomised into ECLS was 180 of 1136 (15.8%) in the intervention group and 176 of 1126 (15.6%) in the control group (OR = 1.016, 95% CI, 0.660 to 1.564). The proportion of patients who positively responded to the invitation was 224 of 1136 (19.7%) in the intervention group and 205 of 1126 (18.2%) in the control group (OR = 1.103, 95% CI, 0.778 to 1.565).

**Conclusions:**

Optimised patient information materials made little difference to the proportion of patients positively responding to a trial invitation or to the proportion subsequently randomised to the host trial.

**Trial registration:**

ClinicalTrials.gov, NCT01925625. Registered on 15 August 2015.

Study Within A Trial, SWAT-23. Registered on 12 April 2016.

**Electronic supplementary material:**

The online version of this article (10.1186/s13063-018-2896-9) contains supplementary material, which is available to authorized users.

## Background

Whilst randomised controlled trials are the gold standard for evaluating the effect of treatments, participant recruitment continues to be the biggest obstacle to their successful delivery [1–3]. In the United Kingdom, increasing numbers of people are approached to participate in trials [4]. Despite this, the proportion of people who actually enrol is small, and recruitment remains a challenge, with between 50% and 80% of all trials not meeting recruitment targets [2, 5, 6]. Poor recruitment into a trial reduces the total sample size (limiting internal validity) and the proportion of eligible participants who are recruited (limiting external validity). Recruitment and retention are now the highest priority for methodological research in academic trials units in the United Kingdom [7], and systematic reviews have highlighted a clear need for recruitment interventions, especially those evaluated in ongoing trials where patients make real (rather than hypothetical) decisions about participation [8–10].

Although proper understanding of the trial is fundamental to valid participant consent, research suggests that trial participants can have insufficient understanding of some aspects, including the burdens and rewards associated with participation as well as their rights to revoke consent once enrolled [11, 12]. Furthermore, at the end of a trial participants may not know the name of the medicine being evaluated [13]. Usually this information is provided in the form of a participant information sheet (PIS); however, PISs are often long and complex, in part to meet the stipulations of research ethics committees. They may also lack visual appeal [14, 15] with suboptimal formatting and writing of the information. These features can adversely affect prospective participants’ willingness to engage with the leaflet and so may go unread. When such leaflets are read, they may affect potential participants’ understanding of a trial, which in turn can negatively impact recruitment (and potentially retention). One way of improving the quality of the PIS is performance-based user testing. This is an iterative process that involves obtaining feedback from the target population for the PIS, expertise in writing for patients and graphic design, and revising the material, which together are aimed at producing an optimised version of participant information materials.

The Systematic Techniques for Assisting Recruitment into Trials (START) study is a research programme, funded by the UK Medical Research Council (MRC) [16], which aimed to increase the evidence base for trial recruitment by developing a platform to advance the rapid and robust evaluation of recruitment interventions. Within START we have developed the methodological and reporting frameworks for embedding recruitment studies within a trial (SWAT) [16, 17], and additionally developed two recruitment interventions (an improved PIS and a multimedia decision aid), which are being evaluated in a series of SWATs in multiple host trials to determine their impact on participant recruitment within individual trials and across different trial contexts [18, 19]. Full details of the MRC START study are provided elsewhere [16].

This article reports the fourth MRC START SWAT developing and evaluating optimised patient information materials (with improved readability and ease of comprehension) in a host trial evaluating a new test for screening lung cancer: the EarlyCDT Lung Cancer Scotland (ECLS) study.

### Objectives

We aimed to evaluate the effectiveness of optimised patient information materials on the numbers of participants responding to the initial invitation to participate and the numbers ultimately enrolled in the ECLS trial.

## Methods

We report the development of the evaluation of the recruitment intervention in line with the guidelines for reporting embedded recruitment SWATs, which adapt Consolidated Standards of Reporting Trials (CONSORT) for recruitment SWATs [17]. The checklist of items for reporting recruitment SWATs is included as Additional file 1.

### Trial design: the ECLS host trial

Lung cancer is the world’s leading cause of cancer-related mortality and a major source of morbidity [20]. ECLS aimed to assess the effectiveness of a new test (EarlyCDT-Lung test) in reducing the incidence of late-stage lung cancer at diagnosis compared with standard clinical practice [21]. Half of those enrolling were randomised to be offered the EarlyCDT-Lung test, a simple blood test to detect seven autoantibodies to aid in the risk assessment and early detection of lung cancer. The other half also had their blood taken, but this was not tested as part of the trial. Intervention participants who had a positive test were followed with an x-ray and serial computed tomographic imaging 6-monthly for 24 months. Control participants received standard care. ECLS aimed to recruit 10,000 participants from Glasgow and surrounding areas in Scotland at the time of the present study. Recruitment into ECLS occurred between August 2013 and August 2016.

In ECLS potentially eligible individuals were identified from general practice (GP) medical records through an electronic medical record search undertaken by the Scottish Primary Care Research Network (SPCRN), which was established in 2002 as a framework to co-ordinate national research activity in primary care. The SPCRN was also responsible for accessing patient details, determining eligibility and mailing trial invitations, which consisted of a GP-signed letter and a participant information booklet. Those responding positively to the invitation could opt into the trial using a posted reply slip, SMS (text) message, email or telephone. Those meeting the trial eligibility criteria and providing consent were recruited. The eligibility criteria were patients aged 50 years to 75 years, willing and able to give informed consent for participation in the trial, and current or ex-smokers with at least a 20-pack-year history (i.e., smoking at least 20 cigarettes per day for 20 years). If patients had less than a 20-pack-year smoking history, they had to have a first-degree relative with a history of lung cancer. The ECLS trial team did not have access to patients’ details until they independently contacted the trial team.

Participants who did not respond to the initial invitation letter were sent a reminder letter, written and designed by the ECLS study team, to determine whether they had received the trial invitation and whether they were interested in taking part. However, this follow-up process was only introduced 7 months after the start of recruitment.

### Trial design: the embedded recruitment SWAT

Recruitment into the SWAT took place over a 5-month period (February–June 2014) until the target sample size of the SWAT was reached. The SWAT adopted a randomised controlled trial design. Patients identified as potentially eligible for the ECLS trial (from GP lists) were individually randomised to either of the following arms:

1. Control participant information brochure (PIB): the original ECLS PIB and covering invitation letter (*see* Additional files 2 and 3)
2. Intervention PIB: the user-tested PIB and invitation covering letter (*see* Additional files 4 and 5).

The recruitment trial included all patients identified as potentially eligible for the ECLS host trial; there were no additional inclusion or exclusion criteria. The ECLS trial team led the implementation of the SWAT in their host trial, with methodological input from the START team. ST was a co-investigator on both ECLS and MRC START and proofread the control PIB in his role on ECLS before START began. ST played no role in the development of the START ECLS participant information leaflet. No other member of the ECLS team was part of the START team.

### Control intervention: PIB

The control PIB was developed by the ECLS host trial team, based at Tayside Clinical Trials Unit (TCTU). This was presented as a booklet of 32 pages in length and approved by The East of Scotland Research Ethics Committee REC1 on 16th April 2013 (reference 13/ES/0024) as part of the ethics application for the ECLS study. Unlike most participant information leaflets in trials, which tend to be written as plain text documents, the control PIB was a coloured document formatted by a professional design company and included photos (Additional file 2). The accompanying GP letter was on a single A4-sized sheet with a tear-off reply slip and contact details on the reverse (Additional file 3).

One of the MRC START investigators (ST) proofread the content of the control PIB in his former role as the assistant director of TCTU and a co-investigator on ECLS. ST’s role in the control PIB did not extend beyond proofreading, with all other work undertaken by the wider ECLS team. None of the other MRC START investigators were involved in the development of the original PIB.

### Recruitment intervention

A revised PIB and accompanying GP letter were developed using the performance-based user-testing process. This was an evidence-based [22], expert-led process that consisted of optimising the readability, appearance and navigation of the PIB and letter. The majority of the content of the original PIB was retained, but the PIB was re-written and re-designed on the basis of feedback from the user-testing process. This process was led by PK from the START team, who has significant experience and expertise in writing for patients, with user testing being undertaken by Luto Research Limited (Leeds, UK). Healthy volunteers with a similar age, educational and employment socio-demographic profile as the sample for ECLS were recruited for the performance-based user testing. Individuals who had participated in any healthcare trial or user testing in the preceding 6 months were excluded. An iterative user-testing process was followed [23–26] which involved objectively evaluating the ability of patients to locate and understand key information contained in the PIB and letter.

Four rounds of user testing were undertaken with ten volunteers in each round. The combined mean age of volunteers across the four rounds was 63 years (range, 51–75 years); 50% were female; 37.5% had completed their education at the UK minimum age (14–16 years, depending on participant age), 42.5% completed education at age 18 years, and 20% had higher education (graduates); and 52.5% were retired, 42.5% were employed and 5% were unemployed. At each round, volunteers were presented with a single version of the PIB and invitation letter, which they were then asked to read. Then each volunteer was asked to find information in the PIB or letter, using 20 structured questions [23, 24]. Seventeen of these questions focused on the PIB, and three focused on the invitation letter. To test the organisation of the information, volunteers were asked to identify the answer in the PIB or letter; to test understanding, they were asked to provide the answer in their own words. The questions focused on the following:

1. The ECLS trial’s nature and aims
2. The process and meaning of consent in ECLS
3. ECLS trial procedures
4. Safety, efficacy and nature of the intervention being evaluated in ECLS

Round 1 involved testing the control ECLS materials, consisting of a 32-page, A5-sized booklet in colour and a two-sided, A4-sized participant invitation letter, which contained an overview of the study on one side and contact details of the ECLS trial team on the other with a tear-off slip. Rounds 2–4 involved versions of the optimised information materials. After rounds 2–4, revisions were made to the materials in response to the obtained user-testing data. If volunteers had difficulty with understanding, it signified a need to revise the wording, and if they had difficulty finding an answer, it signified a need to amend the document’s organisation or navigation. Table 1 below lists the main changes made to the PIB following user testing, which is also attached as Additional file 4. Optimisation of the PIB also involved professional graphic design by a company with significant expertise in designing patient communication materials (Additional file 4).

**Table 1:** Changes to the content and structure of the patient information brochure

Changes to content	Changes to the form and structure
Added NHS Scotland logo to front page	Reduced length from 32 to 30 pages
Shortened ‘foreword’ by 50%, and changed heading to ‘Introduction’	Moved ‘contents’ page from page 4 to page 2, and added trial team contact details at bottom
Changed all but one of the six photographic images to reflect more demographic diversity	Trial team contact details moved from back page to page 27
Added a trial flow chart of the participant pathway in the centre of the booklet	Made contents list clearer and more spread out
Added summary circles of text throughout the booklet	Use of short sentences, plain English, bullet points throughout
New back page, with the same image as front page (map of Scotland) with NHS logo	

The changes to the accompanying invitation letter were as follows: the letter was shortened by removal of content duplicated in the PIB; ‘bullet points’ were added; a 10-point summary of the ECLS trial was printed onto the reverse of the letter; and a tear-off reply slip was placed at the foot of the letter, so that letter text was retained. Additional file 5 shows the changes made to the invitation letter.

### Outcome measures

The primary outcome was the number of patients recruited into the ECLS trial. The secondary outcome was the proportion of patients expressing an interest in participating in ECLS.

### Sample size calculation

The recruitment trial was powered to detect a significant improvement in recruitment rate into ECLS, defined as an absolute increase of 5% above baseline. Baseline response rates for the first five ECLS practices were around 20% (December 2013), although patient ineligibility and difficulties contacting some people reduced the 20% response rate to a recruitment rate of approximately 14% in later practices. For a baseline of 20% recruitment, a sample size of approximately 2000 patients was estimated to provide 80% power and alpha of 0.05 for a 5% minimally important increase in recruitment between the intervention PIB and the control PIB.

### Randomisation

Potential participants identified from GP lists as eligible were randomly allocated to receive the control PIB or intervention (user-tested PIB) and GP covering invitation letter at a 1:1 ratio using the recruitment-tracking software developed by the Health Informatics Centre, University of Dundee, and the TCTU.

### Statistical methods

Analyses were conducted in line with a standard statistical plan developed at Barts and the London Pragmatic Clinical Trials Unit. We initially described outcomes separately by arm for patients who expressed an interest in the study and those who were recruited into ECLS. We then compared these using logistic regression. Analyses followed the intention-to-treat principle and were conducted using Stata version 14 software (StataCorp, College Station, TX, USA). An independent statistician (VM) who conducted analyses remained blind to allocation until the analyses were complete.

## Results

**Fig. 1:**
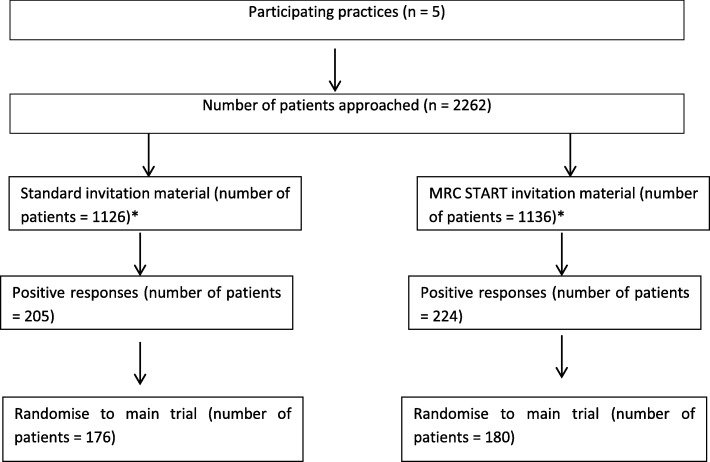
Flow chart of participant response and recruitment. Based on the guidelines for reporting embedded recruitment trials, which adapt Consolidated Standards of Reporting Trials (CONSORT) for embedded recruitment trials [17]. *MRC START* Medical Research Council Systematic Techniques for Assisting Recruitment to Trials study

A total of 2262 patients were randomised for the SWAT, of whom 1136 were sent the intervention PIB and 1126 were sent the control PIB. For the primary outcome, the proportion of patients enrolled and randomised into ECLS (the host trial) was 180 of 1136 (15.8%) for those sent the intervention PIB and 176 of 1126 (15.6%) in the control PIB group (OR = 1.016; 95% CI, 0.660 to 1.564). Figure 1 outlines the recruitment flow chart for the SWAT. For the secondary outcome, the proportion of patients who responded positively to the invitation and expressed an interest in trial participation was 224 of 1136 (19.7%) for patients sent the intervention PIB and 205 of 1126 (18.2%) in the control PIB group (OR = 1.103; 95% CI, 0.778 to 1.565).

### Harms

We did not measure potential harms, such as perceptions of increased pressure to participate among patients receiving the intervention PIB.

## Discussion

### Summary of main findings

We evaluated the effectiveness of optimised patient information materials on improving recruitment into a lung cancer screening trial. Being sent the optimised patient information materials made little difference to the proportion of patients positively responding to a trial invitation or to the proportion being randomised.

### Strengths and limitations

We systematically developed optimised patient information materials on the basis of an established and published process [23–26], according to a published protocol [16], and we report our findings in line with best practice guidance for reporting recruitment SWATs [17]. This SWAT was fully powered and used an a priori sample size calculation, unlike most SWATs, including those conducted as part of the MRC START project, which set sample sizes on the basis of convenience [27]. In line with the statistical analysis plan, we undertook the analysis according to the initial randomisation. However, although in the SWAT all the initial invitations were correctly sent as per random allocation to the intervention or control PIB, the ECLS team sent out further reminders in two practices to patients who had not responded, in the intervention as well as in the control arm of the SWAT, which were not optimised or randomised. The use of these reminders was a capacity decision, and at the time of the SWAT, not all patients were sent reminders; this was because sending out more fresh invitations through newly recruited GPs led to a better recruitment return than reminding non-responders. However, because the reminder letters were not randomised, their use may have diluted the effect of the recruitment intervention, although this effect is mitigated because we aimed to identify differences in proportions between the intervention and control groups, rather than absolute levels. This highlights some of the issues with undertaking recruitment SWATs, including difficulties in aligning the SWAT and host trials [28].

A limitation of our study is that we were unable to gather data to assess any moderators of the effect of the intervention, such as age, gender, ethnicity or socioeconomic status, which may have provided additional information on the impact of the intervention in different groups. It was not the aim of the study to undertake qualitative interviews with patients sent the trial information, so we were not able to explore the wider impact of optimised patient materials beyond recruitment rates. There are also a number of different ways in which the intervention PIB was optimised (we were evaluating a particular way of producing a PIB, rather than any single change to the PIB), thus in the absence of a process evaluation or a series of trials of individual PIB changes, it is difficult to determine whether any single change or different combination of changes may have been more effective. The user-testing process may have had a positive impact in its own right by improving readability and ease of comprehension and therefore may have led to better engagement with the trial; however, we did not assess this.

In comparison with the materials used in many clinical trials, the original PIB was of high quality, with use of colour photographs and developed by a highly experienced trials team. Additionally, the original PIB was proofread by an ECLS co-investigator who was a START co-investigator. Thus it may not have been representative of the typical PIB developed by trial teams. This may have limited the potential additional benefit of the user-testing process and may explain the lack of difference between the intervention and control groups. However, our present findings with an OR of 1.016 (95% CI, 0.660 to 1.564) are in line with those of the three other SWATs undertaken as part of START, where the ORs of the user-tested versus control leaflets were as follows: 1.01 (95% CI, 0.71–1.45) [29], 1.12 (95% CI, 0.78 to 1.61) [18] and 1.63 (95% CI, 1.00 to 2.67) [18]. Therefore, all current SWATs to date have found little or no effect of the intervention PIB compared with the control PIBs, suggesting that proofreading of the control PIB by a START co-investigator did not significantly impact the control leaflet in this current SWAT. The latest Cochrane review also undertook a meta-analysis of these SWATs with an overall risk difference estimate of 1% (95% CI, − 1% to 3%). The START ECLS risk difference is 0% (95% CI, − 3% to 3%) and so is entirely consistent with the other three. Trialists now routinely involve patients and the public to assist with developing information leaflets for patients, which may reduce the relative benefits of user testing.

In this SWAT ‘harm’ could include reduced recruitment in the intervention PIB group. We therefore evaluated a two-tailed hypothesis for the primary and secondary outcomes, which accepted that sending the recruitment intervention to potential participants could cause benefit or loss to recruitment for the host trial. Although patients not being recruited represent a loss to the host trial, for the patient, not being enrolled in the trial may not be harmful, because the patient may have made an informed decision not to participate. The results demonstrate that the recruitment intervention was not effective for increasing response and randomisation rates.

### Comparison with existing literature

This SWAT adds to the small but emerging literature on the effects of modified information on trial recruitment. In the Cochrane review of recruitment interventions [9], three trials explored the impact of supplementary written material on recruitment and found little evidence of benefit. As part of the MRC-START programme, this article reports the fourth SWAT evaluating the effects of optimised participant information materials on trial recruitment in different trial contexts [18, 29]. This will enable us to determine the effectiveness of the intervention within each individual host trial and across different trial contexts and patient populations, using a meta-analysis. We are taking this approach because recruitment interventions may have different impacts according to the specific contexts, trial interventions and patient populations. In this specific SWAT, we tested the intervention in the context of a screening trial. Previous SWATs of the same intervention have been undertaken in a falls prevention trial [29], and in two trials delivering telehealth interventions for patients with cardiovascular disease and depression [18]. These trials have shown small increases in the numbers of patients positively responding and enrolling; however, such increases were not statistically significant. This SWAT shows similar results in a small but statistically non-significant increase in response and recruitment rates. In this SWAT, the proportions of patients responding in both intervention (19.7% response; 15.8% enrolled) and control (18.2% response; 15.6% enrolled) groups were higher than in our previous trials; for example, the Healthlines Depression recruitment SWAT achieved recruitment rates of 6.3% in the intervention group versus 4% in the control group (OR = 1.63; 95% CI, 1.00 to 2.67) [18]. This may have been a consequence of the use of reminder letters in both the intervention and control groups. All current SWATs of this enhanced PIB intervention have been compared with original PIBs developed by highly experienced host trial teams based within UK Clinical Research Collaboration-accredited Clinical Trials Units, which have some of the most experienced teams delivering trials in the United Kingdom. It may be that the optimised leaflets may be found to be more effective if compared with leaflets developed by less experienced trial teams in the United Kingdom or elsewhere.

### Implications for recruitment research

As part of the START programme we have undertaken a series of SWATs of optimised participant information materials to determine their overall effectiveness within individual trials and across different trial contexts. In START we have demonstrated the feasibility of developing and evaluating recruitment interventions in multiple ongoing trials. Future research should focus on reducing uncertainty around the effect of existing interventions used to improve recruitment (such as telephone reminders) and developing and evaluating new interventions to support trial recruitment, especially those interventions targeting the education and training of trial recruiters, which has been highlighted as a priority topic around recruitment into trials [30, 31]. Improving the evidence base around recruitment has the potential to increase recruitment rates and increase the proportion of trials delivering on time.

## Conclusions

We evaluated the effectiveness of optimised patient information materials on recruitment into a trial of a screening test for lung cancer. Optimised patient information materials did not increase the proportion of patients positively responding or being randomised. This SWAT adds to the evidence base around trial recruitment and will contribute to a future meta-analysis of the effectiveness of optimised information materials as part of the MRC-funded START project and as part of the Cochrane systematic review of recruitment interventions, which is led by a member of our team. Further interventions addressing identified priorities for recruitment research should be developed and evaluated using SWATs.

## Additional files

Additional file 1: Checklist of items for reporting embedded recruitment trials, based on the guidelines for reporting embedded recruitment trials, which adapts Consolidated Standards of Reporting Trials (CONSORT) for embedded recruitment trials. (DOCX 17 kb)

Additional file 2: Original patient information brochure. (DOCX 1004 kb)

Additional file 3: Original accompanying GP letter. (DOCX 558 kb)

Additional file 4: Optimised patient information brochure. (DOCX 1720 kb)

Additional file 5: Optimised accompanying GP cover letter. (DOCX 434 kb)

## Abbreviations

ISRCTN: International Standard Randomised Controlled Trial Number
MRC START: Medical Research Council Systematic Techniques for Assisting Recruitment to Trials
NHS: National Health Service
NRES: National Research Ethics Service
PIB: Patient information brochure
PIS: Patient information sheet
SPCRN: Scottish Primary Care Research Network
