# Supplementary material for: The effect of optimised patient information materials on recruitment in a lung cancer screening trial: an embedded randomised recruitment trial
Source: Trials. 2018 Sep 18;19:503. doi: 10.1186/s13063-018-2896-9 (PMC6145341; doi:10.1186/s13063-018-2896-9)
Supplement: Supplementary file 1 — Checklist of items for reporting embedded recruitment trials, based on the guidelines for reporting embedded recruitment trials, which adapts Consolidated Standards of Reporting Trials (CONSORT) for embedded recruitment trials. (DOCX 17 kb) [file 13063_2018_2896_MOESM1_ESM.docx]

**Checklist of items for reporting embedded recruitment trials. Based on the ‘guidelines for reporting embedded recruitment trials’, which adapts Consolidated Standards of Reporting Trials (CONSORT) for embedded recruitment trials.**

| **Section/topic and item no.** | **Extension for embedded recruitment trials** | **Reported on page no.** |
| --- | --- | --- |
| 1a | Identification as an *embedded randomised recruitment trial* in the title | 1 |
| 1b | Structured summary of *embedded recruitment trial* design, methods, results, and conclusions (for specific guidance see CONSORT for abstracts) | 3 |
| 2a | Scientific background and explanation of rationale for the *embedded recruitment trial including a brief description of the host trial(s) as appropriate* | 4 |
| 2b | Specific objectives or hypotheses for the *embedded recruitment trial* | 5 |
| 3a | Description of *embedded recruitment trial* design (such as parallel, factorial, *cluster*) including allocation ratio | 5 |
| 3b | Important changes to methods of the *embedded recruitment trial* after commencement (such as eligibility criteria), with reasons | N/A |
| 4a | Eligibility criteria for participants for the *embedded recruitment trial, including any differences from those for the host trial(s)* | 6 |
| 4b | Settings and locations where the *embedded recruitment trial was carried out, including a brief description of the host trial(s) as appropriate* | 5 |
| 5 | The interventions for each group *(including control group) within the embedded recruitment trial* with sufficient details to allow replication, including how, where and when they were actually administered | 6-7 |
| 6a | Completely defined pre-specified primary and secondary outcome measures for the *embedded recruitment trial,* including how and when they were assessed | 7 |
| 6b | Any changes to *embedded recruitment* trial outcomes after the *embedded recruitment trial* commenced, with reasons | N/A |
| 7a | How sample size for the *embedded recruitment trial* was determined | 7 |
| 7b | When applicable, explanation of any interim analyses and stopping guidelines for the *embedded recruitment trial* | N/A |
| 8a | Method used to generate the random allocation sequence for the *embedded recruitment trial* | 7 |
| 8b | Type of randomisation; details of any restriction (such as blocking and block size) in the *embedded recruitment trial* | 7 |
| 9 | Mechanism used in the *embedded recruitment trial* to implement the random allocation sequence (such as sequentially numbered containers), describing any steps taken to conceal the sequence until interventions were assigned | 7 |
| 10 | Who generated the random allocation sequence(s), who enrolled participants, and who assigned participants to *embedded recruitment* interventions? | 7 |
| 11a | If done, who was blinded after assignment to *embedded recruitment* interventions (for example, participants, care providers, those assessing outcomes) and how? | 8 |
| 11b | If relevant, description of the similarity of interventions in the *embedded recruitment trial* | N/A |
| 12a | Statistical methods used to compare groups for primary and secondary outcomes of the *embedded recruitment trial* | 7-8 |
| 12b | Methods for additional analyses, such as subgroup analyses and adjusted analyses for the *embedded recruitment trial* | 7-8 |
| 13a | For each group in the *embedded recruitment trial,* the numbers of participants who were randomly assigned, received intended treatment, and were analysed for the primary outcome | 8 |
| 13b | For each group, losses and exclusions after randomisation to the *embedded recruitment trial,* together with reasons | 8 |
| 14a | Dates defining the periods of recruitment and follow-up *for both embedded recruitment trial and host trial(s)* | 5 |
| 14b | Why the *embedded recruitment trial* ended or was stopped | 5 |
| 15 | *If possible* a table showing baseline characteristics *of each arm of the embedded recruitment trial* | *N/A* |
| 16 | For each group in the *embedded recruitment trial,* number of participants (denominator) included in each analysis and whether the analysis was by original assigned groups | 8 |
| 17a | For each primary and secondary outcome, results for each group in the *embedded recruitment trial,* and the estimated effect size and its precision (such as 95 % confidence interval) | 8 |
| 17b | For binary outcomes in the *embedded recruitment trial*, presentation of both absolute and relative effect sizes is recommended | 8 |
| 18 | Results of any other analyses performed for the *embedded recruitment trial*, including subgroup analyses and adjusted analyses, distinguishing pre-specified from exploratory | 8 |
| 19 | All important harms or unintended effects in each group*for both the embedded recruitment trial and host trial(s)*(for specific guidance see CONSORT for harms) | 8 |
| 20 | *Embedded recruitment trial* limitations, addressing sources of potential bias, imprecision, and, if relevant, multiplicity of analyses | *8-9* |
| 21 | Generalisability (external validity, applicability) *of the embedded recruitment trial* findings | 8-10 |
| 22 | Interpretation consistent with results *of the embedded recruitment trial,* balancing benefits and harms, and considering other relevant evidence | 8-10 |
| 23 | Registration number and name of trial registry *(for all host trials and embedded recruitment trial if available)* | 3 |
| 24 | Where the *embedded recruitment trial* protocol can be accessed, if available | 4 |
| 25 | For the *embedded recruitment trial,* sources of funding and other support, role of funders *and collaborators* | 11 |
