# Supplementary figures and images for: The effect of optimised patient information materials on recruitment in a lung cancer screening trial: an embedded randomised recruitment trial
Source: Trials. 2018 Sep 18;19:503. doi: 10.1186/s13063-018-2896-9 (PMC6145341; doi:10.1186/s13063-018-2896-9)

## Additional file 2: Original patient information brochure


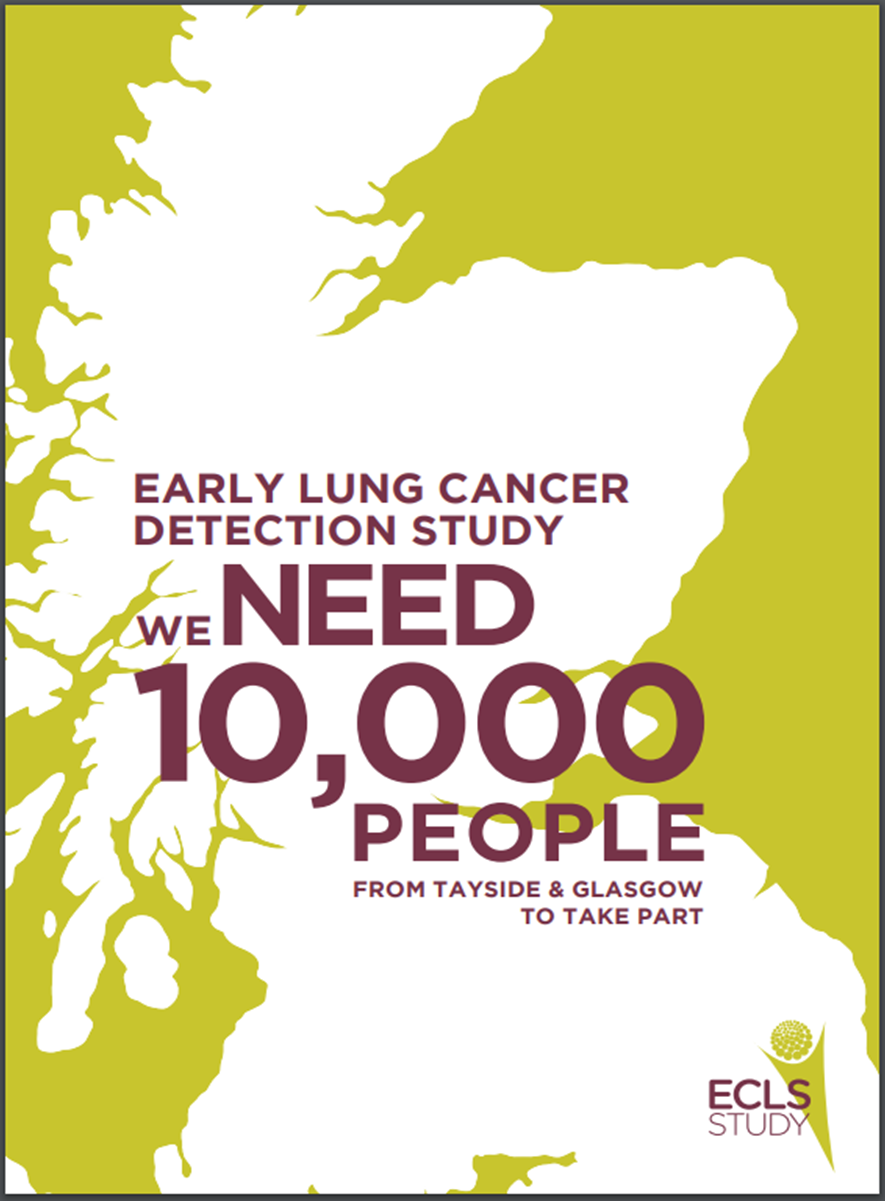


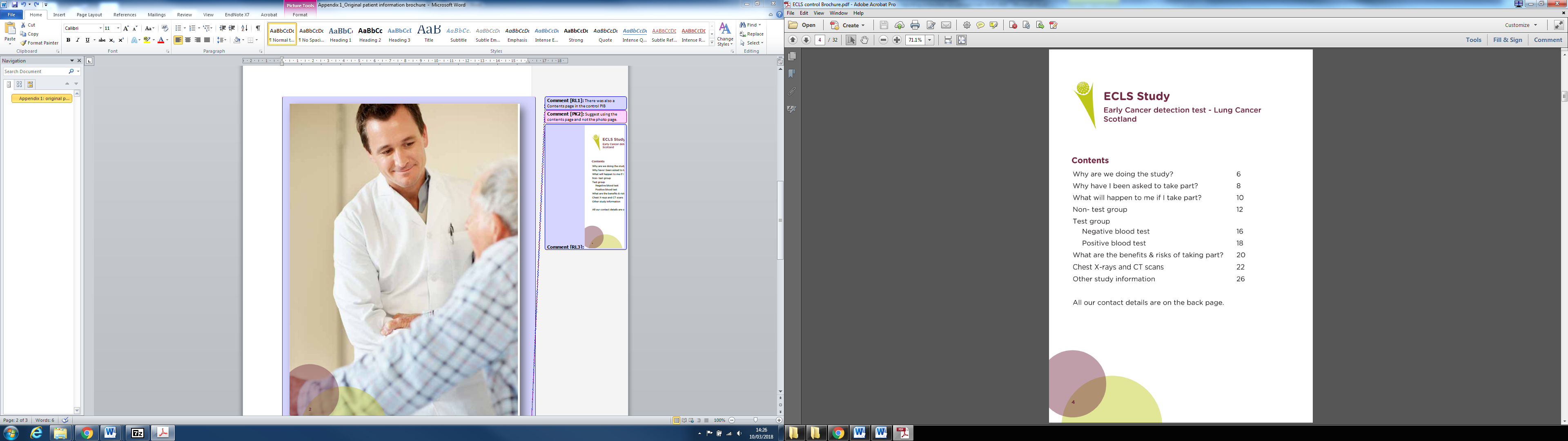

Supplement: Supplementary file 2 — Original patient information brochure. (DOCX 1004 kb) [file 13063_2018_2896_MOESM2_ESM.docx]

## Additional file 3: Original accompanying GP letter


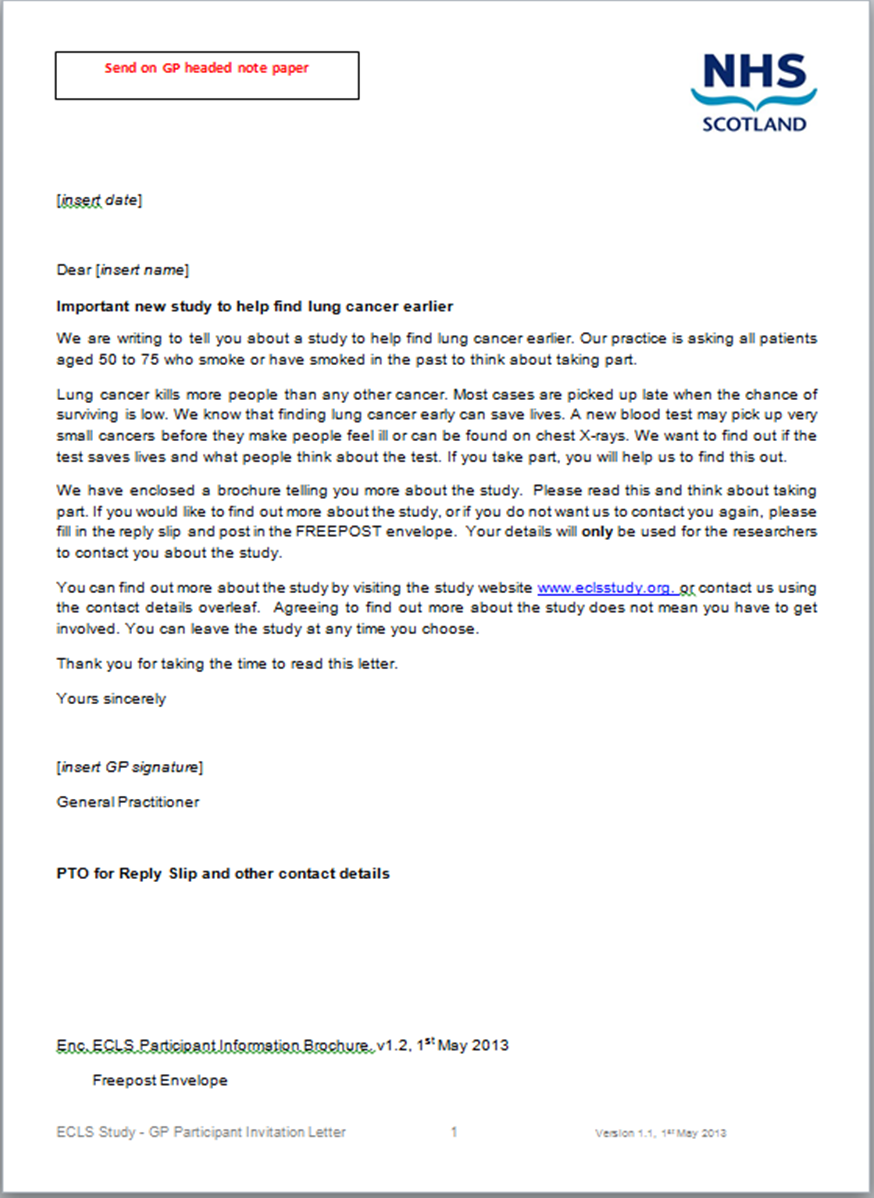


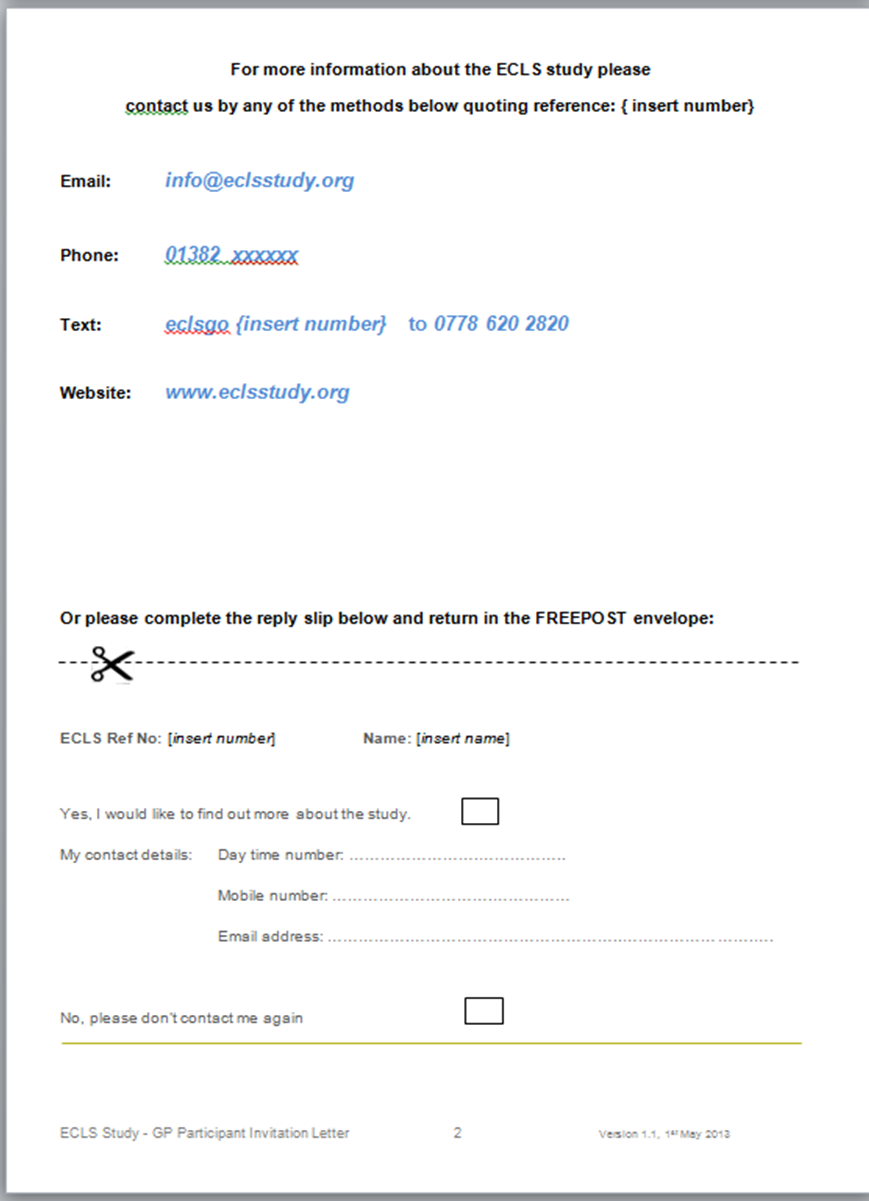

Supplement: Supplementary file 3 — Original accompanying GP letter. (DOCX 558 kb) [file 13063_2018_2896_MOESM3_ESM.docx]

## Additional file 4: Optimised patient information brochure


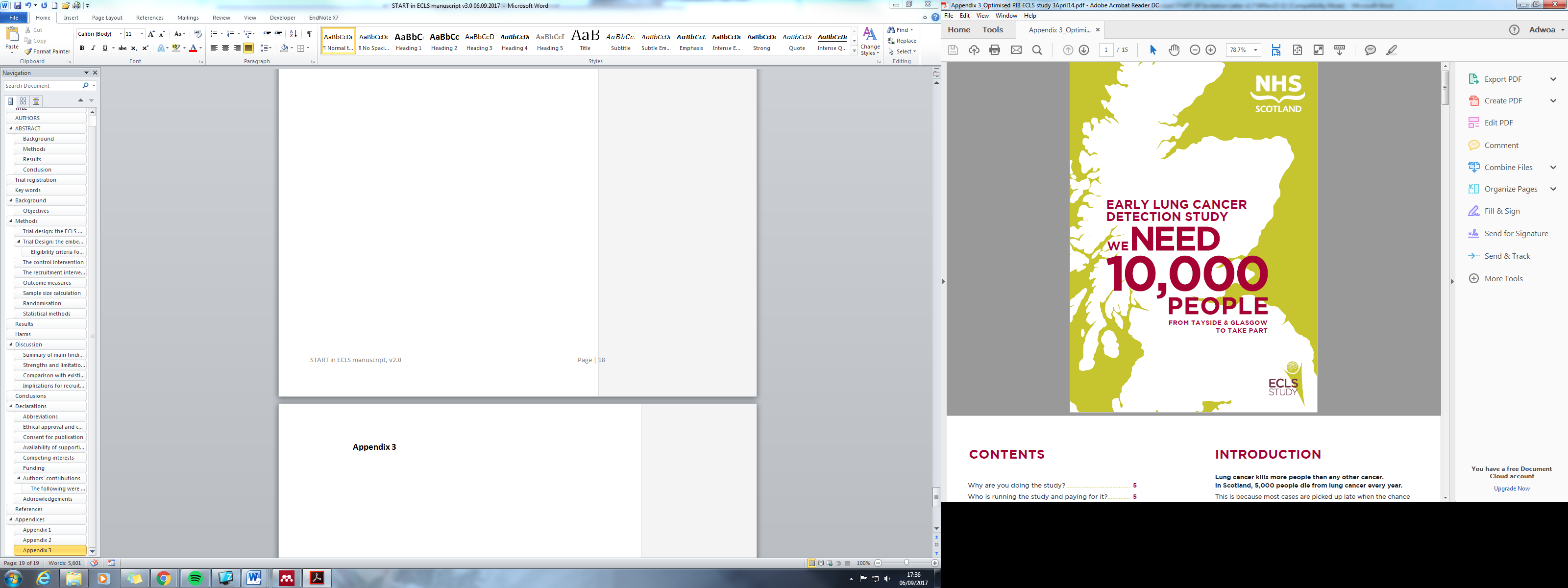


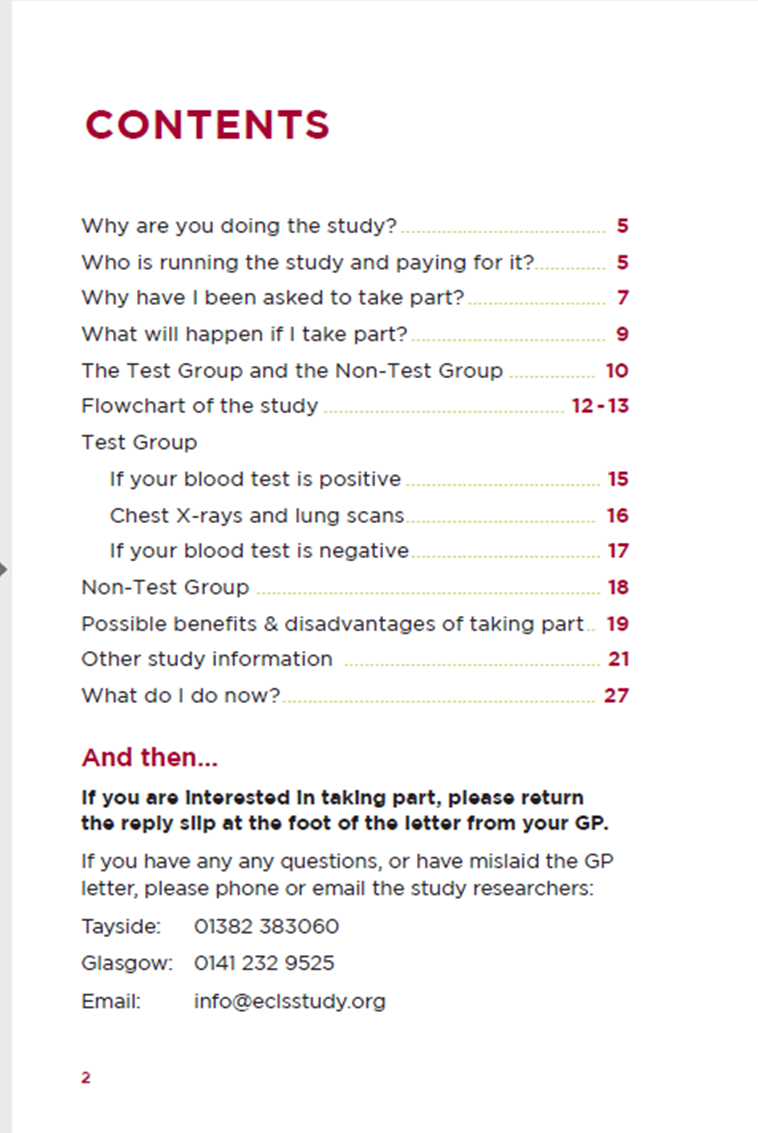


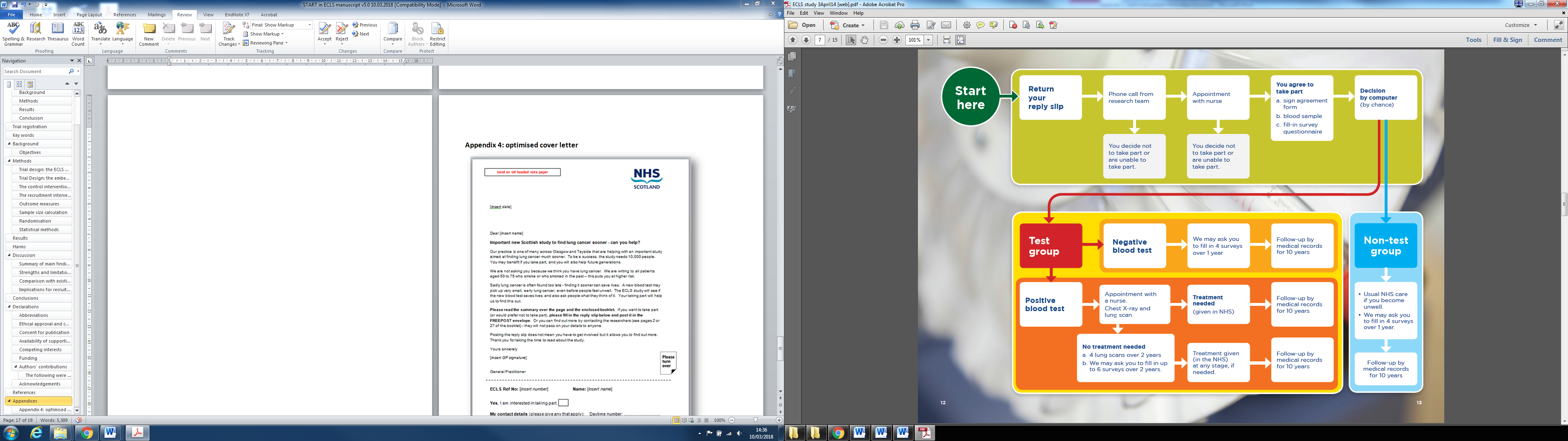

Supplement: Supplementary file 4 — Optimised patient information brochure. (DOCX 1720 kb) [file 13063_2018_2896_MOESM4_ESM.docx]
