# Supplementary material for: The effect of optimised patient information materials on recruitment in a lung cancer screening trial: an embedded randomised recruitment trial
Source: Trials. 2018 Sep 18;19:503. doi: 10.1186/s13063-018-2896-9 (PMC6145341; doi:10.1186/s13063-018-2896-9)
Supplement: Supplementary file 5 — Optimised accompanying GP cover letter. (DOCX 434 kb) [file 13063_2018_2896_MOESM5_ESM.docx]

## Additional file 5: optimised GP cover letter
